# *Pogostemon cablin* Triggered ROS-Induced DNA Damage to Arrest Cell Cycle Progression and Induce Apoptosis on Human Hepatocellular Carcinoma In Vitro and In Vivo

**DOI:** 10.3390/molecules25235639

**Published:** 2020-11-30

**Authors:** Xiao-Fan Huang, Gwo-Tarng Sheu, Kai-Fu Chang, Ya-Chih Huang, Pei-Hsiu Hung, Nu-Man Tsai

**Affiliations:** 1 Institute of Medicine, Chung Shan Medical University, Taichung 40201, Taiwan; s9870509@gmail.com (X.-F.H.); gtsheu@csmu.edu.tw (G.-T.S.); kfchang1015@gmail.com (K.-F.C.); hwangjy319@gmail.com (Y.-C.H.); 2 Department of Medical Laboratory and Biotechnology, Chung Shan Medical University, Taichung 40201, Taiwan; 3 Director of Traditional Chinese Medicine, Ditmanson Medical Foundation Chia-Yi Christian Hospital, Chiayi 60002, Taiwan; cych05086@gmail.com; 4 Department of BioIndustry Technology, Da-Yeh University, Changhua 51591, Taiwan; 5 Clinical Laboratory, Chung Shan Medical University Hospital, Taichung 40201, Taiwan

**Keywords:** hepatocellular carcinoma (HCC), *Pogostemon cablin* (PPa extract), cell cycle, apoptosis, synergism, chemoprevention

## Abstract

The purpose of the study was to elucidate the anti-hepatoma effects and mechanisms of *Pogostemon cablin* essential oils (PPa extract) in vitro and in vivo. PPa extract exhibited an inhibitory effect on hepatocellular carcinoma (HCC) cells and was less cytotoxic to normal cells, especially normal liver cells, than it was to HCC cells, exerting a good selective index. Additionally, PPa extract inhibited HCC cell growth by blocking the cell cycle at the G_0_/G_1_ phase via p53 dependent or independent pathway to down regulated cell cycle regulators. Moreover, PPa extract induced the FAS-FASL-caspase-8 system to activate the extrinsic apoptosis pathway, and it increased the bax/bcl-2 ratio and reduced ΔΨm to activate the intrinsic apoptosis pathway that might be due to lots of reactive oxygen species (ROS) production which was induced by PPa extract. In addition, PPa extract presented to the potential to act synergistically with sorafenib to effectively inhibit HCC cell proliferation through the Akt/mTOR pathway and reduce regrowth of HCC cells. In an animal model, PPa extract suppressed HCC tumor growth and prolonged lifespan by reducing the VEGF/VEGFR axis and inducing tumor cell apoptosis in vivo. Ultimately, PPa extract demonstrated nearly no or low system-wide, physiological, or pathological toxicity in vivo. In conclusion, PPa extract effectively inhibited HCC cell growth through inducing cell cycle arrest and activating apoptosis in vitro and in vivo. Furthermore, PPa extract exhibits less toxicity toward normal cells and organs than it does toward HCC cells, which might lead to fewer side effects in clinical applications. PPa extract may be developed into a clinical drug to suppress tumor growth or functional food to prevent HCC initiation or chemoprotection of HCC recurrence.

## 1. Introduction

Hepatocellular carcinoma (HCC) is the third leading cause of cancer death worldwide [1]. In addition, HCC has a poor prognosis because of chronic hepatitis, with cirrhosis leading to the deterioration of liver function. Moreover, intrahepatic metastasis result in highly recurrence [2]. Sorafenib is generally acknowledged as the standard of care to improve the overall survival (OS) of patients with advanced HCC. Though sorafenib improves the OS of patients with HCC, the clinical benefit is transient, and the toxicity as well as poor antitumor effects of sorafenib remain unsolved issues. With increasing advances in medicine, the combination of chemotherapy agents remains a promising therapeutic strategy for increasing the response rate of advanced HCC patients, for instance, regorafenib and bevacizumab and so on. Another type of agent that is attracting considerable interest is immune checkpoint inhibitors, such as anti-PD-1/PD-L1 (Nivolumab, Pembrolizumab) or CTLA-4 antibodies, and phase III studies of such inhibitors are currently under investigation [3]. Thus, there is obviously a need for effective therapeutic options for HCC patients. Furthermore, new strategies are needed not only to prevent the development or posttreatment recurrence of HCC but also to enhance survival or quality of life [4,5].

Herbal medicine is considered a great way to improve therapeutic efficacy and reduce toxic effects. In the past, many chemotherapeutic agents have been derived from natural products with effective therapeutic effects or low toxicity in treating various illnesses [6,7]. A large number of herbal products have been used worldwide to manage many kinds of liver diseases because of their safety, curative effects and minimal adverse effects. In addition, a number of studies have shown that medicinal herbs function via several mechanisms, such as suppressing carcinogenesis, inhibiting oxidative injury, and reducing inflammation, which protect the normal function of the liver [8]. Hence, the development of new pharmacologically effective chemotherapeutic agents from natural plants that can trigger cancer cell death would be a significant clinical benefit.

*Pogostemon cablin* has been orally and topically administered in Asia for centuries as a pharmaceutical product for curing exogenous fever, headache, hypotension, allergy, thirst, ache, dysentery, diarrhea, and inflammation [9]. Scientific studies have revealed that *Pogostemon cablin* has the following biological activities: antidepressant [10,11], antimicrobial [12,13], antiviral [14], anti-inflammatory [15], gastroprotective [16,17], antiaging [18], and antitumor activities [19,20]. Moreover, *Pogostemon cablin* has been found to inhibit colon cancer proliferation through the induction of cell cycle arrest at the G_0_/G_1_ phase. However, it is still unknown what the role of *Pogostemon cablin* is in hepatocellular carcinoma, and the molecular mechanisms behind its anti-hepatoma activity are also unclear.

Our group has demonstrated that *Pogostemon cablin* essential oils which abbreviated form of PPa extract in this study induced apoptosis in human hepatocellular carcinoma HepG_2_ cells through oxidative stress-regulated mitochondrial dysfunction involving the p53/p21 and apoptotic pathways. Based on the findings of previous work, we investigated the role of apoptosis in the anticancer effect of PPa extract in HepG_2_ cells in vitro and the underlying mechanisms of apoptosis-related signaling pathways. These findings demonstrated for the first time that PPa extract induced apoptosis by activating the caspase cascade, and we revealed the underlying antitumor effects of PPa extract plus sorafenib in vitro. These results could provide novel insights into the mechanisms underlying the anticancer effects of PPa on human hepatoma cells.

## 2. Results

### 2.1. PPa Extract Inhibited HCC Cell Growth

**Figure 1:**
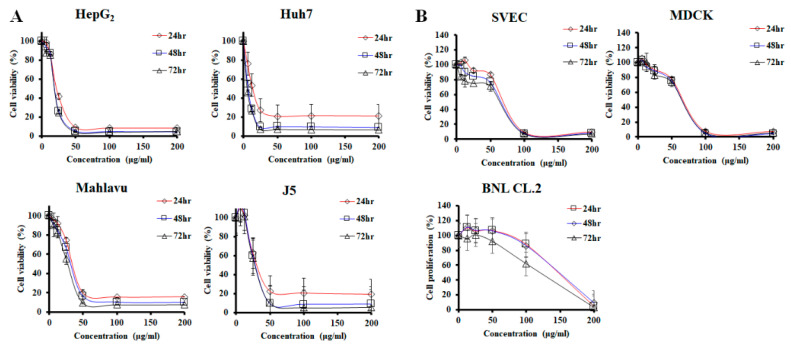
PPa extract inhibited HCC cell growth with less toxicity to normal cells. (**A**) Cell viability of HCC cells after PPa extract treatment (0–200 μg/mL), as assessed by MTT assay. (**B**) The viability of normal cells treated with PPa extract (0–200 μg/mL). The results are presented as the mean ± SD.

To address the effect of PPa extract on cell proliferation in human hepatoma cells, cells were treated with PPa extract at increasing doses over various periods of time. As shown in Figure 1A, treatments with increasing concentrations of PPa extract over increasing periods of time decreased the cell viability from 100% to 5%, showing that PPa extract inhibited HCC cell proliferation in a dose-dependent manner. The PPa extract showed a 50% inhibition at concentrations ranging from 7.34 ± 3.09 to 33.29 ± 2.72 μg/mL in hepatoma cells. Moreover, 5-FU, VP-16 and sorafenib are known for their inhibitory effects on hepatoma cells, and the IC_50_ of those drugs ranged from 1.77 ± 4.31 to 18.79 ± 0.91 μg/mL in hepatoma cells (Table 1). To further determine the effects of PPa extract on the growth of normal cells which were in a non-proliferative state, the results showed that PPa extract possessed less inhibitory ability than normal cells (Figure 1B). The IC_50_ of PPa extract on SVEC and MDCK cells was 69.68 ± 4.63 and 73.61 ± 0.16 μg/mL, respectively. Interestingly, the IC_50_ of PPa extract in a normal liver cell type, BNL CL.2 cells, was 147.24 ± 7.71 μg/mL, indicating that PPa extract apparently exhibited a smaller inhibitory effect on normal cells. After that, to explore the selective index (SI), which is defined greater than 2 presenting good selectivity [21], Table 2 shows that the PPa extract exhibited better SI values (2.1–20.1) than sorafenib (1.4–6.1) and VP-16 (0.4–2.1). Consequently, PPa extract demonstrated inhibitory effects on hepatoma cells but exerted less cytotoxic effects on normal cells. In addition, the PPa extract exhibited a good selective index, suggesting that the PPa extract might possess fewer side effects than other agents.

### 2.2. PPa extract Altered the Cell Cycle Distribution in HCC cells

**Figure 2:**
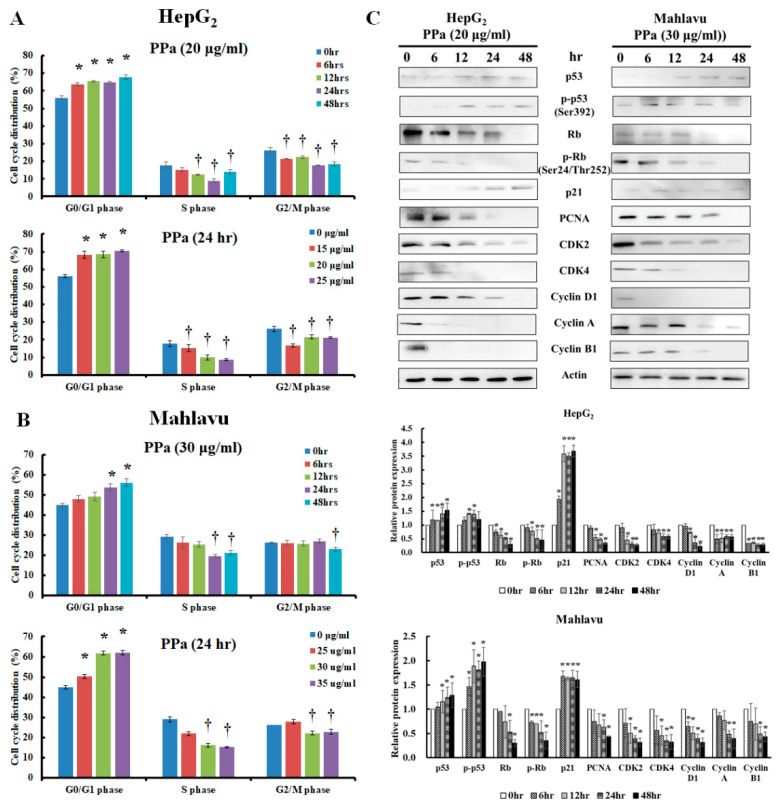
PPa extract blocked the cell cycle at G_0_/G_1_ phase in HCC cells. (**A**,**B**) HepG_2_ and Mahlavu cells were treated with PPa extract for the indicated time intervals, and the cell cycle distribution was analyzed by flow cytometry. (**C**) After PPa extract treatment, cell lysates were collected and analyzed for cell cycle regulators by Western blotting with the indicated antibodies. *, †: Significant difference between the control group and experimental group, *p* < 0.05.

PPa extract was next studied to determine if it altered the cell cycle distribution to exert its inhibitory effect on hepatoma cells. The results indicated that while PPa extract treatment of HepG_2_ cells did increase the number of cells in G_0_/G_1_ phase (from 57% to 71%), it also decreased the number of cells in S and G_2_/M phase (from 16% to 3%; from 26% to 17%) (Figure 2A). Similar results were observed in Mahlavu cells, and the cell population in the G_0_/G_1_ phase increased from 45% to 62%; however, it decreased the cell population of the S and G_2_/M phase (from 29% to 15%; from 26% to 22%) (Figure 2B). The results showed that both cell lines induced cell cycle arrest at the G_0_/G_1_ phase. Hence, we aimed to gain insight into the changes in tumor suppressors and cell cycle regulators. After PPa extract treatment in HepG_2_ cells, PPa extract induced the expression of p53 and *p*-p53 proteins, and it increased the expression of p21 protein, resulting in decreased levels of the following downstream proteins: PCNA, cdk4, cdk2, cyclin D1, cyclin A, and cyclin B1. Mahlavu cells with a p53 mutant were exposed to PPa extract treatment, and the results showed that the expression of p-p53 was not obviously changed; however, the expression of p21 was modest increased, and the expression of downstream proteins decreased, suggesting that PPa extract also induced a p53-independent pathway and subsequently upregulated the expression of p21 (Figure 2C). Moreover, PPa extract also decreased the expression of total Rb and p-Rb in both cell lines. Taken together, the data revealed that PPa extract induced cell cycle arrest at the G_0_/G_1_ phase through induction of p53-dependent and p53-independent pathways to increase the expression level of p21, leading to the decreased expression of cell cycle regulators.

### 2.3. Ppa Extract Stimulated ROS Production and Imbalanced Mitochondrial Membrane Potential in HCC Cells

**Figure 3:**
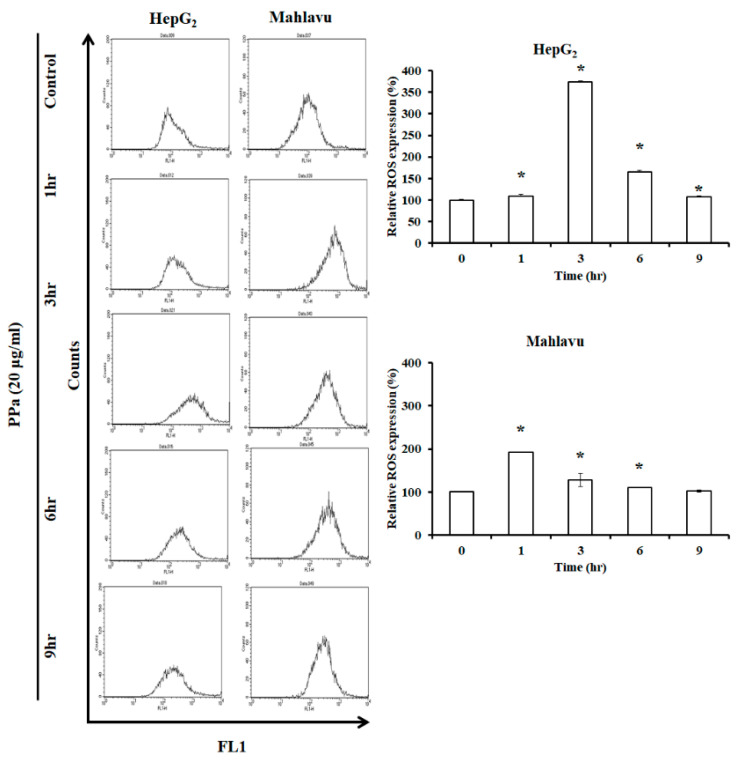
PPa extract induced ROS generation. HepG_2_ and Mahlavu cells were treated with PPa extract (20 μg/mL) for time intervals and analyzed by flow cytometry at FL1-H to detect ROS production. *: Significant difference between the control group and experimental group; *p* < 0.05.

**Figure 4:**
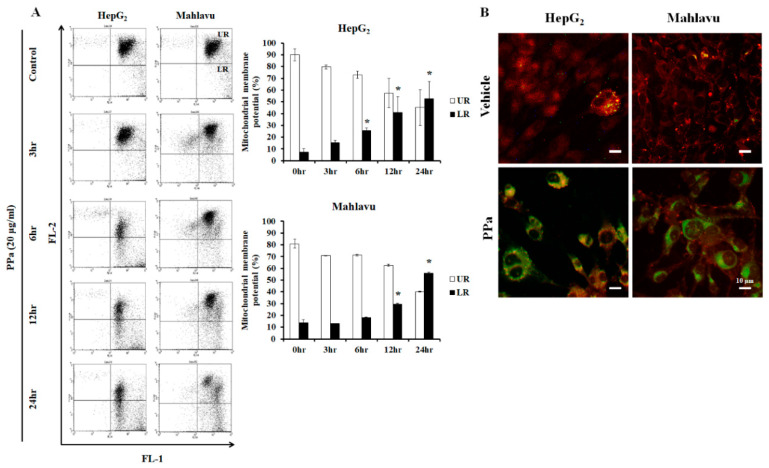
PPa extract stimulated mitochondrial membrane potential loss. (**A**) Mitochondrial membrane potentials (MMPs) were measured at FL2-H and FL1-H channels after PPa extract treatment in HCC cells. (**B**) After PPa extract treatment, JC-1 fluorescence was detected. *: Significant difference between the control group and experimental group; *p* < 0.05.

Former results revealed that p53/p21 pathway was activated to arrest cell cycle at G_0_/G_1_ phase. The high concentration of ROS production can damage DNA, causing activation of p53 (Ser392), which is phosphorylated by DNA damage signaling [22], and consequently increases p21 expression. Then, ultimately, cell cycle arrest will occur to repair damaged DNA for subsequent cell cycle proceed. Moreover, cells exposure to high dose of ROS may cause severe damage to DNA, proteins and lipids, and cells will arrest in all phases of the cell cycle and will undergo apoptosis. Consequently, we wondered whether PPa extract induced ROS production in PPa extract-treated HepG_2_ and Mahlavu cells and the results found that PPa extract rapidly increased the ROS level within 3 h in both HCC cell lines (Figure 3). Further, ROS can also induce mitochondrial dysfunction resulting in mitochondrial membrane potential (MMPs, ΔΨm) imbalance that can activate intrinsic apoptosis pathway. Further, ΔΨm was evaluated, and the results showed significantly increased ΔΨm (by 30–40%) within 12 h of treatment in HepG_2_ and Mahlavu cells (Figure 4A). Moreover, after PPa extract treatment, JC-1 fluorescence was observed, and the results showed that green fluorescence was increased, suggesting that PPa extract induced mitochondrial membrane potential (ΔΨm) loss (Figure 4B).

### 2.4. PPa Extract Induced Extrinsic and Intrinsic Apoptosis in HCC Cells

**Figure 5:**
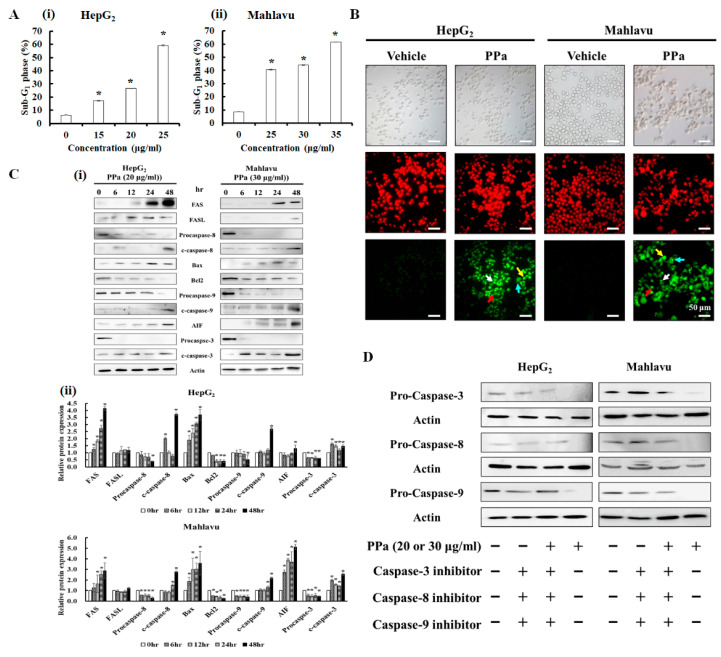
PPa extract induced HCC cell apoptosis by activating both the extrinsic and intrinsic apoptotic pathways. (**A**) After PPa treatment, the sub-G_1_ phase of the cell population was analyzed by flow cytometry; (i): HepG_2_ cells; (ii): Mahlavu cells. (**B**) HepG_2_ and Mahlavu cells were incubated with PPa extract for 24 h and analyzed by TUNEL assay. TUNEL positive (green); PI: propidium iodide (red); red arrow: chromatin condensation; yellow arrow: DNA fragments; blue arrow: anoikis; and white arrow: apoptotic bodies. (**C**) Western blots for pro-apoptotic and anti-apoptotic proteins in PPa extract-treated HCC cells; (i): indicated protein expressions; (ii): the quantitative data of protein expressions. (**D**) Western blots for caspase-3, -8 and -9 proteins in HepG_2_ and Mahlavu cells which pretreated with caspase-3, -8 or -9 inhibitors (1 μM) for 2 h and then treated with PPa extract (20 or 30 μg/mL). *: Significant difference between the control group and experimental group; *p* < 0.05.

Subsequently, the percentage of PPa extract-treated HCC cells that died was detected by flow cytometry. HepG_2_ and Mahlavu cells were exposed to serial doses PPa extract for 24 h, and the data showed that in both cell types PPa extract increased the number of cells in the sub-G_1_ phase in a dose-dependent pattern (Figure 5A). Then, TUNEL assays were used to determine whether PPa extract induced apoptosis. As shown in Figure 5B, after PPa extract treatment, the two cell lines exhibited cell shrinkage under light field microscopy, and the number of TUNEL-positive cells increased, as indicated by anoikis, DNA fragmentation, chromatin condensation and apoptotic body formation. To further elucidate the PPa extract-induced apoptosis pathway in HCC cells, Western blotting was utilized. After exposure to PPa extract, the expression of FAS and FASL increased, the expression of procaspase-8 decreased, and cleaved caspase-8 increased, indicating that the extrinsic apoptotic pathway might be activated (Figure 5C). Furthermore, detecting Bax/Bcl2 ration was increased, resulting in the downregulation of procaspase-9 and cleaved caspase-9 increasing, suggesting that the intrinsic apoptosis pathway might be activated. Additionally, the expression of AIF increased after PPa extract treatment, suggesting that PPa extract might also induce the caspase-independent apoptosis pathway to cause cell death. And then, the expression of procaspase-3 was decreased and cleaved caspase-3 was increased, revealing that the caspase cascade might be involved. After that, to confirm that PPa extract activated the caspase cascade, HepG_2_ and Mahlavu cells were pretreated with caspase-3, -8, or -9 inhibitors (1 μM) for 2 h. Then, the cells were treated with PPa extract (20 μg/mL) for 24 h and were analyzed by Western blot. The results revealed that PPa extract indeed induced extrinsic as well as intrinsic apoptosis pathway activation, which activated the caspase cascade (Figure 5D). These results validated that PPa extract activates the caspase cascade via extrinsic and intrinsic apoptosis pathways, leading to HCC cell death. Taken together, our data showed that PPa extract contributed to the production of ROS and triggered p53/p21 expression to affect mitochondrial membrane potential (ΔΨm), resulting in the activation of the apoptosis pathway.

### 2.5. Synergistic Inhibitory Effects Induced by PPa Extract Plus Sorafenib in Hepatoma Cells

**Figure 6:**
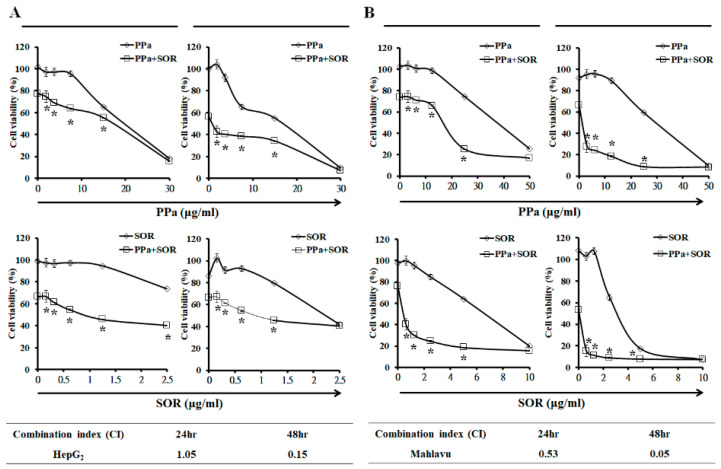
PPa extract synergized with sorafenib to enhance the inhibitory ability of PPa extract on HCC cell growth. (**A**), (**B**) HepG_2_ and Mahlavu cells were treated with one drug or a combination of drugs and then were evaluated by the combination index. SOR: sorafenib. *: Significant difference between the control group and experimental group; *p* < 0.05.

**Figure 7:**
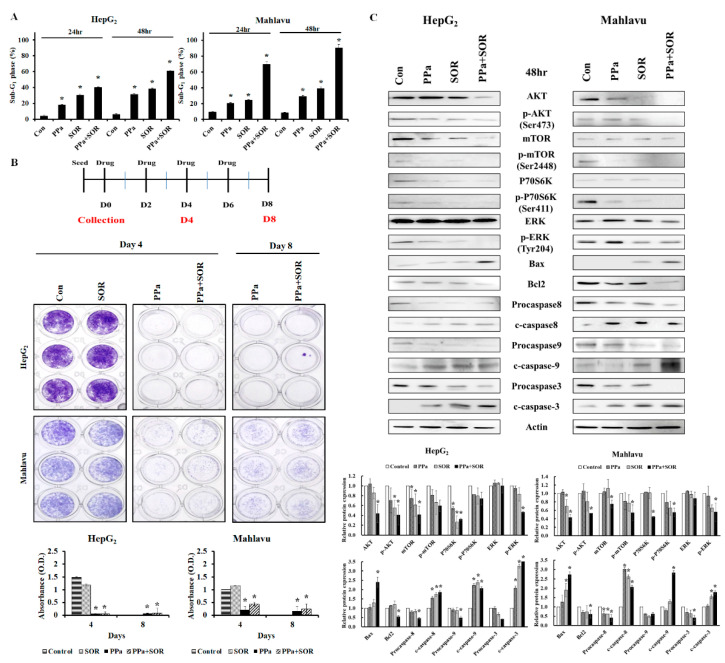
PPa extract plus sorafenib reinforced the suppression of cell proliferation and induction of cell apoptosis in HCC cells. (**A**) HepG_2_ cells were treated with 2 μg/mL sorafenib plus 15 μg/mL PPa extract, and Mahlavu cells were treated with 3 μg/mL sorafenib plus 25 μg/mL PPa extract for 24 and 48 h. After combined treatment, both cell lines were analyzed for the percentage of sub-G_1_ phase by flow cytometry. (**B**) HepG_2_ and Mahlavu cells were treated with PPa extract (15 μg/mL) and sorafenib (0.2 μg/mL) for 4 and 8 days. After treatment, cells were stained with crystal violet and absorbance was measured at 550 nm to calculate cell viability. (**C**) HepG_2_ and Mahlavu cells were treated with combined treatment for 48 h. Cell extracts were prepared and analyzed by Western blotting with the indicated antibody. CON: control; SOR: sorafenib. *: Significant difference between the control group and experimental group; *p* < 0.05.

To examine synergism between PPa extract and sorafenib, HepG_2_ and Mahlavu cells were treated in combination with indicated concentrations of drugs to calculate the combination index (CI). As shown in Figure 6, the results revealed that the combination of the PPa extract plus sorafenib exerted a synergistic effect in HepG_2_ cells at 48 h, and significant synergy was observed in Mahlavu cells at 24 and 48 h. Both cell lines showed CI values of less than 1 at 48 h, and Mahlavu cells with the p53 mutant were more sensitive to PPa extract plus sorafenib. Next, we addressed the effects of the two drugs in combination on the induction of cell death by observing the sub-G_1_ cell population. Indeed, HepG_2_ and Mahlavu cells appeared to be sensitive to PPa extract plus sorafenib, resulting in a marked increase in the sub-G_1_ cell population (Figure 7A). In addition, to assess the ability of PPa extract plus sorafenib to inhibit cell regrowth at day 4 and day 8. The results showed sorafenib no inhibitory effect at 0.2 μg/mL concentration on day 4 and PPa extract continuedly showed antiproliferation effects on both of cells. PPa extract combined with sorafenib showed inhibitory effects in both cell lines, indicating that the two-agent combination could prevent HCC cell regrowth (Figure 7B). Furthermore, to elucidate the antiproliferation mechanism of the two drugs, the AKT/mTOR, ERK and caspase cascades signaling pathway was examined. First, PPa extract used in combination with sorafenib reduced AKT, pAKT (Ser473), mTOR, p-mTOR (Ser2448), P70S6K, and p-P70S6K (Ser411) expression, suggesting that the combination might inhibit HCC cell growth by suppressing the AKT/mTOR signaling pathway. Second, ERK/pERK expression was also detected, and the results revealed that PPa extract plus sorafenib resulted in a more dramatic reduction in p-ERK (Tyr204) protein expression than what was observed in controls. These results revealed PPa extract plus sorafenib suppressed the expression of AKT/mTOR and ERK signaling in HepG_2_ and Mahlavu cells. Then, to test the combination of PPa extract and sorafenib on induction of apoptosis, the results showed the combination of PPa extract and sorafenib was found to strongly reduce the protein expression of pro-caspase-8, -9, and -3. In converse, cleaved caspase-8, -9, and-3 protein expressions and Bax/Bcl2 ratio were significantly increasing., revealing that the combination of these two drugs enhanced the induction of cell apoptosis in HepG_2_ and Mahlavu cells (Figure 7C). These results suggested that PPa extract in combination with sorafenib exhibited a synergistic effect that reduced HepG_2_ and Mahlavu cell proliferation and regrowth via the induction of cell death and inhibition of the AKT/mTOR and ERK pathway.

### 2.6. PPa Extract Suppressed Hepg_2_ Tumor Growth and Exhibited Less Toxicity in HCC Xenograft Model

**Figure 8:**
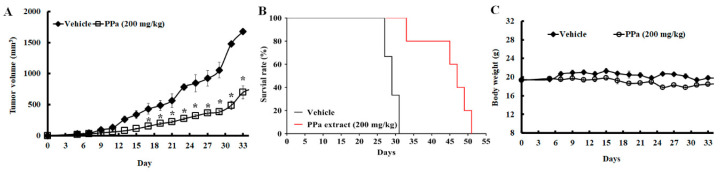
PPa extract suppressed HCC cell proliferation in the xenograft model. Balb/c nude mice were injected with HepG_2_ cells on day 0 of the experiment, started treatment after five days and then were treated with PPa extract (200 mg/kg) every two days. When the tumor volume reached 1500 mm^3^, the mice were sacrificed. (**A**) Tumor volume. (**B**) Survival rates. The data are expressed as the mean ± SEM. *: Significant difference between the control group and experimental group, *p* < 0.05. (**C**) Balb/c nude mice were injected with HepG_2_ cells, which was followed by administration of PPa extract (200 mg/kg) and measurement of body weight.

**Figure 9:**
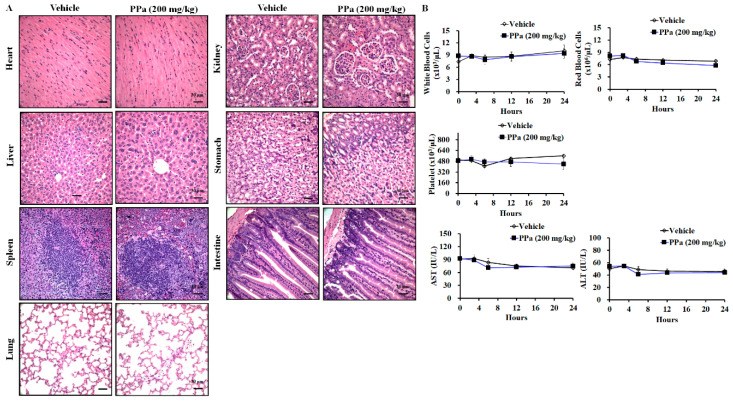
PPa extract displayed low pathological and physiological toxicity in vivo. (**A**) After sacrificing the animals, organs were collected and analyzed by HE staining. (**B**) After PPa treatment, blood was collected at the 0, 3, 6, 12, and 24 h for analysis of blood cells (white blood cell, red blood cell and platelet) and serum biochemistry (ALT, and AST).

To further assess the inhibitory effect of PPa extract on growth was evaluated in HCC xenografts in nude mice. As shown in Figure 6, Balb/c nude mice bearing xenograft tumors were administered PPa extract (200 mg/kg, subcutaneous injection once every two days). Volumes of tumors and body weights of mice were measured every two days during the experimental period. The results revealed that PPa extract exerted greater antitumor effects than vehicle treatment (Figure 8A). In addition, we found that PPa extract prolonged the lifespan of mice by a range of 31 days to 51 days (Figure 8B). As shown in Figure 8C, we found no significant differences in body weight between vehicle- and PPa extract-treated mice. These results revealed that PPa extract exerted an antihepatoma capacity to suppress HCC tumor growth and extended survival time with no remarkability changes of body weight in vivo. As we had monitored the body weights throughout the study, PPa extract did not dramatically decrease the body weight, revealing that PPa extract might not cause severe systemic toxicity in vivo. Subsequently, we further assess the pathology of the following organs: heart, liver, spleen, lung, kidney, stomach, and intestine, after PPa extract treatment. Notably, the cell morphology of these organs did not appear obviously change and remained the integral structure of organs (Figure 9A). Further, no significant the blood and immune cell infiltration were observed after PPa extract administration. These results revealed that PPa extract might not cause severe organ damage to recruit immune cells and activate inflammation. Moreover, we found no significant differences in the WBC, RBC, and platelet counts (Figure 9B). Importantly, the values of AST and ALT showed no significant differences when comparing the control with PPa extract treatment groups, revealing that PPa extract might not further cause severe liver cell damage and toxicity, which lead to liver dysfunction. The data demonstrated that PPa extract presented a lower physiological and pathological toxicity in vivo.

### 2.7. PPa Extract Induced Apoptosis and Reduced Autocrine Proliferation in Xenograft Model

**Figure 10:**
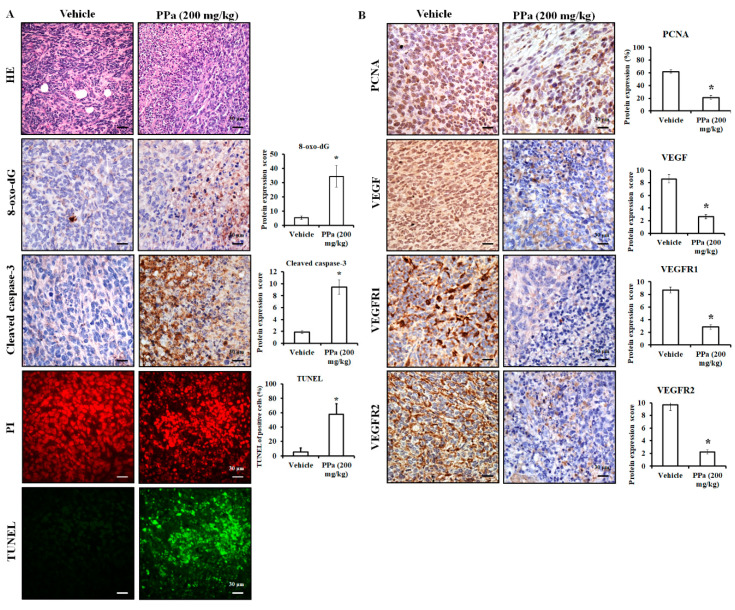
PPa extract affected ROS generation, cell apoptosis and proliferation. After the mice were sacrificed, the tumor mass was collected for HE and IHC analysis. (**A**) After PPa extract treatment, tumor cell damage, 8-oxo-dG, as well as cleaved caspase-3 were observed and TUNEL assay was performed to detect apoptosis in HCC tissue. PI: propidium iodide (red); TUNEL: green. (**B**) Autocrine proliferative proteins were examined by IHC staining. *: Significant difference between the control group and experimental group; *p* < 0.05.

**Figure 11:**
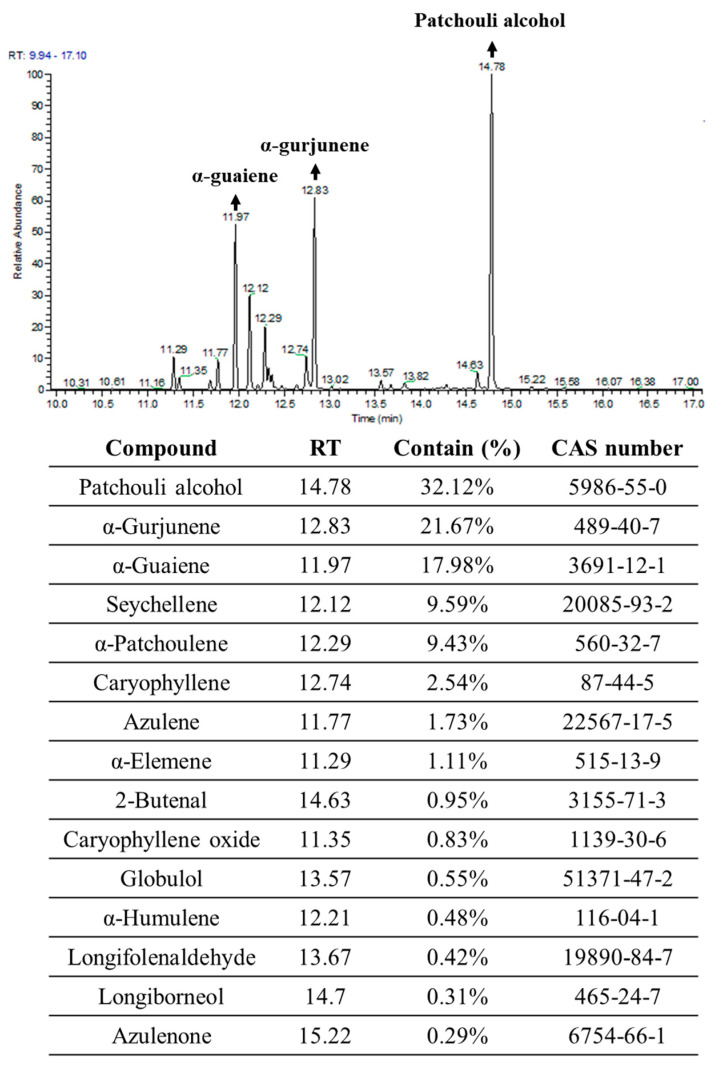
GC/MS analysis of PPa extract. Gas chromatography-mass spectrometry (GC-MS) analyses were performed by the National Central Taiwan University Office of Research and Development’s Center for Advanced Instrumentation (Hsinchu, Taiwan). Components were identified by comparing their mass spectra with those obtained from authentic samples or spectra of the Wiley/Nist libraries. RT: Retention time.

Next, we addressed the inhibitory effect of PPa extract in vivo with H&E and IHC staining. The results showed that PPa extract caused HCC tumor cell death due to the induction of ROS production and causing DNA damage in tumor cells, leading to an increase in 8-oxo-dG expression, which is the commonly used marker of oxidative stress-derived DNA damage [23,24]. (Figure 10A). Additionally, PPa extract increased the expression of cleaved caspase-3 and TUNEL positive (green) lead to apoptosis in vivo. These results indicated that PPa extract induced ROS production to damage tumor cells causing activation of apoptosis in vivo that was consistent with the funding in vitro. As shown in Figure 10B, PPa extract also suppressed the expression of PCNA, VEGF, VEGFR1 and VEGFR2, resulting in inhibition of HCC growth. In conclusion, PPa extract exhibited inhibitory effects on HCC through ROS production, induction of apoptosis, and suppression of autocrine proliferation. Ultimately, to elucidate the components of PPa extract, we utilized GC/MS analysis. The data revealed that patchouli alcohol (RT: 14.78; 32.12%), α-gurjunene (RT: 12.83; 21.67%) and α-guaiene (RT: 11.97; 17.98%) were three major components in the PPa extract and there were other components, including seychellene, α-patchoulene, caryophyllene, azulene, α-elemene, 2-butenal, caryophyllene oxide, globulol, α-humulene, longifolenaldehyde, longiborneol, and azulenone (Figure 11). Among these components, the content of patchouli alcohol was highest, and this indicated that patchouli alcohol might be the active ingredients to exert antihepatoma capacity.

## 3. Discussion

Hepatocellular carcinoma (HCC) is the most frequent tumor and the third most common malignancy, and it causes high mortality worldwide; in addition, the incidence of HCC has been increasing. Although many treatment approaches have been used to treat advanced HCC, for now, only transarterial chemoembolization (TACE) and sorafenib have been shown to provide survival benefit [2]. As a result, better options for the prevention of HCC development might be a good approach. Here, we used *Pogostemon cablin*, a plant of the Lamiaceae family that is native to tropical regions of Asia, and studies have demonstrated many biofunction activities of *Pogostemon cablin*, including antidepressant [10,11], antimicrobial [12,13], antiviral [14], anti-inflammatory [15], gastroprotective [16,17], antiaging [18], and antitumor activities [19,20]. Among these, our previous study has demonstrated the anticancer activity of *Pogostemon cablin* extract on colon cancer in vitro and in vivo. In the study, *Pogostemon cablin* extract induces apoptosis and cell cycle arrest and presented no obvious pathological toxicity in vivo [20]. These results indicate that *Pogostemon cablin* extract is a potential anticancer agent for cancer treatment. As a result, our study explored the potential role of PPa extract in inhibiting human hepatocellular carcinoma. Here, we first demonstrated that PPa extract inhibited HCC cell proliferation, which was shown in the following cells: HepG_2_ cells—a cell line with WT p53 derived from a patient from the United States [25]; Mahlavu cells—a cell line with mutated p53 with poor differentiation derived from a patient from Africa; Huh7 cells—a cell line with mutated p53 derived from a Japanese patient [26]; and J5 cells—a cell line derived from Taiwan patients. The p53 gene is the most commonly mutated tumor suppressor gene in various human cancers, and hepatocellular carcinoma is no exception [27,28]. Moreover, mutations in p53 are a poor prognostic indicator for survival, suggesting that patients with p53 mutations have a worse prognosis than those with WT p53 [29]. Therefore, PPa extract effectively repressed HCC cell growth and might provide an option for increasing patient benefit. Moreover, the IC_50_ values of *Pogostemon cablin* extract were variety on different types of colon cancer cells and HCC cells, revealed that its’ anticancer potential on these two cancers. On the other hand, HCC patients are common to have a different level of liver dysfunction that restricts the use of chemodrugs result in the limitation of therapeutic efficacy. Herein, present study had to assess inhibitory effect of PPa extract in normal cells including SVEC, MDCK and BNL CL.2 cells and the results revealed that the IC_50_ values of tumor cells compared with normal cells were ranged from 2.1 to 20.1 in PPa extract treatment. These results demonstrated that PPa extract might have less cytotoxicity to normal cells, including epithelial cells, kidney cells and liver cells; moreover, the AST as well ALT values remained in normal range after PPa extract treatment. These results revealing that PPa extract might not induce severe side effects and adverse liver dysfunction.

Subsequently, we further examined the inhibitory effects of PPa extract on alteration of cell cycle distribution because of the cell cycle as an important mechanism for controlling cell growth. The results showed that PPa extract induced cell cycle arrest at the G_0_/G_1_ phase in both HepG_2_ and Mahlavu cells in a dose- and time-dependent manner. After that, the regulation of cell cycle progression was explored. p53 as well as Rb is a tumor suppressor and plays a crucial role in governing the cell cycle and apoptosis [30]. The p21 is a downstream protein of p53 and a CKI that can block the cell cycle at the G_0_/G_1_ phase and G_2_/M transitions by inhibiting cdk4,6/cyclin D and cdk2/cyclin E, respectively. Our results showed that PPa extract induced p53 and p-p53 protein expression to increase downstream proteins p21 expression and reduced the level of cdk2, ckd4, and cyclin D1, A, and B1, resulting in cell cycle arrest at the G_0_/G_1_ phase in WT p53 cells (HepG_2_) and mutant p53 cells (Mahlavu). Moreover, p21 can bind to PCNA to inhibit DNA replication [31], and the data revealed that the expression of PCNA was decreased after PPa extract. The Rb promotes E2F-dependent gene expression to stimulate DNA replication and proceed G_1_/S phase transition [32]. Our results showed that PPa extract reduced Rb and p-Rb expression, which decreased DNA replication to block the G1/S transition lead to cell cycle arrest in HepG_2_ and Mahlavu cells.

Exogenous or endogenous ROS can activate p53 phosphorylation at Ser392 by directly damaging of nuclear and mitochondrial DNA [22] and stimulate transcription of proapoptotic genes that including intrinsic and extrinsic apoptosis pathway, such as Bax, Fas, and FasL [33,34]. Moreover, cytosolic p53 enhances mitochondrial membrane depolarization by causing rearrangement of Bax/Bak on outer mitochondrial membrane and subsequent release apoptotic factors, such as AIF which is involved in DNA fragmentation and activate caspase independent apoptosis. Then, apoptosome complex is formed to activated caspase-9 and effector caspases such as caspase-3 leading to intrinsic apoptosis [35,36]. ROS can directly activate extrinsic apoptosis pathway and recruits cleaved caspase-8 and -3 to trigger apoptosis [37]. As a result, we next to detect the ROS generation of PPa extract-induced in both HepG_2_ and Mahlavu cells and the results indicated that PPa extract induced abundant ROS production contributing to mitochondrial membrane potential imbalance. Meanwhile, sub-G_1_ phase was observed to increase after PPa extract treatment, and TUNEL positive data revealed that PPa extract induced cell apoptosis with classical cell death morphology, including anoikis, DNA fragments, chromatin condensation and apoptotic bodies. To gain insight into the mechanisms of the PPa extract-induced apoptosis pathway, extrinsic, intrinsic, and caspase-independent associated proteins were detected. Fas and FasL are recognized as major pathways involved in the cleavage of caspase-8, which induces extrinsic apoptosis. Moreover, some studies have revealed that HCC cells show resistance to apoptosis because of their suppression of FAS expression [38], and serum levels of soluble FASL in patients with hepatocellular carcinoma show potential as a clinical parameter to evaluate prognosis [39]. Our results showed that PPa extract enhanced FAS and FASL protein expression to induce HepG_2_ and Mahlavu cell apoptosis via extrinsic apoptosis. On the other hand, p53 can directly activate several genes, including Bax, Bcl-2 and AIF. Increasing the Bax/Bcl-2 ratio and AIF expression participate in the regulation of apoptotic mitochondrial membrane permeabilization, resulting in the induction of caspase-9 cleavage and activation of the intrinsic apoptosis pathway. Therefore, PPa extract induced mitochondrial membrane potential loss via both the intrinsic apoptosis pathway and AIF regulation. AIF also plays an important role in inducing caspase-independent chromatin condensation and DNA fragmentation [40]. Consequently, our results found that PPa extract stimulated lots of ROS generation and activated p-p53 (Ser392) expression, which is correlated with DNA damage, leading to caspase dependent apoptosis (extrinsic and intrinsic) and caspase independent apoptosis pathway. Furthermore, these results showed that PPa extract exerted a similar anticancer activity on colon cancer cells and HCC [20].

Drug resistance and hepatotoxicity are important considerations during HCC treatment. Moreover, some clinical studies assessed the combination of standard therapies with traditional herbal medicine and observed significant survival benefits, such as a reduction in recurrence and prolonged survival time [41,42]. Hence, we found that PPa extract seemed to be a good adjuvant for sorafenib, which worked synergistically in both HepG_2_ and Mahlavu cells by enhancement of inhibition cell viability, significantly. Moreover, previous statistical reports indicate that the HCC recurrence rate accounted for 20–25% per year after therapeutic procedure [43]. Our results demonstrated that PPa extract plus sorafenib also diminished the regrowth of HepG_2_ and Mahlavu cells, suggesting that PPa extract combined with sorafenib might provide new approaches for posttreatment chemoprevention regimes. Furthermore, our former data showed that PPa extract stimulated abundant ROS production to repress HCC cell growth and contribute to cell death. Further, sorafenib is reported that it can lead to HCC cell death by inducing of ROS production in vitro and in vivo [44]. ROS can activate PI3K, inactivate PTEN that negatively regulates the synthesis of PIP3 and inhibits the activation of AKT [45]. Moreover, deregulation of the AKT/mTOR pathway has increasingly been implicated in HCC [46], and activation of AKT is thought to mediate the resistance of sorafenib [47]. Additionally, the ERK pathway may also be involved in chemodrugs-induced drug resistance in HCC [48]. Then, to examine the synergistic mechanisms of PPa extract plus sorafenib and the results revealed that the combinational treatment reinforced to reduce the AKT/mTOR pathway and level of ERK, indicated that PPa extract plus sorafenib might be induce more ROS production than drug alone to repress HCC cell growth and reduce the potential of sorafenib-resistant development on AKT/mTOR and ERK pathway. Furthermore, abundant ROS that generated by PPa extract plus sorafenib caused more cell death through induction of caspase dependent pathway. Therefore, the results showed a lower concentration of drugs was needed to induce more cell death and enhance the inhibitory ability by blocking the AKT/mTOR pathway. These results provided a new therapeutic approach for clinical utilization of PPa extract. Moreover, our previous data regarding the anticancer activity of *Pogostemon cablin* extract on colon cancer cells have also shown its capacity for combination with 5-FU [20], and these results might indicate that *Pogostemon cablin* extract probably would be good adjuvant for cancer treatment.

PPa extract induced ROS generation to affect p53/p21 expression, cell cycle-associated regulators, downstream death ligands/receptors and mitochondrial-mediated apoptosis pathways in vitro. Then, we had PPa extract administration in animal model and PPa extract effectively suppressed HCC tumor growth and prolonged the lifespan of mice. Subsequently, considering system toxicity and more specific toxicity, such as hepatoxicity or nephrotoxicity, is extremely important. Additionally, advanced hepatocellular carcinoma is commonly characterized by liver dysfunction attributable to the presence of chronic liver disease and cirrhosis. During PPa extract administration, we measured body weight every two days, and the differences in body weight between the groups appeared to be not statistically significant, suggesting that PPa extract might exhibit few systemic toxic effects in vivo. After two months of treatment, organ toxicity was evaluated, and no obvious pathological damage was observed, suggesting that PPa extract might not have no obviously accumulated toxicity to normal organs. Moreover, the numbers of WBCs, RBCs and platelets were not distinctly different from those in the vehicle group, suggesting that PPa extract might not be induce strong inflammation and blood lysis. In addition, ALT and AST were in normal ranges in both vehicle and PPa treatments, revealing that PPa extract presented little or low liver toxicity in vivo. Taken together, PPa extract might not be induce strong adverse effects in vivo and could be developed as an adjuvant or chemodrug on HCC treatment.

Subsequently, in HCC xenograft, PPa extract demonstrated that it stimulated ROS production, triggered level of cleaved caspase-3 and lead to HCC cell apoptosis that was consistent with the results we observed in vitro. On the other hand, many studies have revealed that HCC cells produce and secrete VEGF and express VEGFR to promote tumor proliferation, indicating the activity of VEGF/VEGFR autocrine and paracrine signaling pathways in HCC cells [49,50]. Moreover, VEGF/VEGFR signaling is positively correlated with tumor size, intrahepatic metastasis, vascular invasion, and TNM stage, which affect prognosis and survival time [51]. Hence, the VEGF/VEGFR signaling axis is an ideal target for treating HCC. After PPa extract administration, VEGF, VEGFR1 and VEGFR2 expression was reduced in HCC tumor tissue, suggesting that PPa extract effectively decreased the VEGF/VEGFR signaling axis to mitigate the autocrine and paracrine signaling pathways. Consequently, PPa extract induced p53-dependent or p53-independent signaling activation to trigger p21 protein expression and block cell cycle progression via downregulation of cell cycle regulators and reduction of DNA replication by inhibition of PCNA and Rb expression in vitro. In addition, PPa extract modulated the VEGF/VEGFR signaling axis to inhibit HCC tumorigenesis in vivo. Ultimately, the ingredient of PPa extract was analyzed by GC/MS and patchouli alcohol, α-gurjunene, and α-guaiene were three major components in PPa extract. However, our previous study showed that *Pogostemon cablin* extract from Republik Indonesia contained several compounds, including azulene, α-guaiene, patchouli alcohol, α-patchoulene, and γ-gurjunene [20]. We assumed that *Pogostemon cablin* from different origin might be the reason why it had different chemical composition. Among these, patchouli alcohol has been reported the anticancer activity on lung and colon cancer [52,53]. Patchouli alcohol presented anticancer effect by inhibiting of histone deacetylases (HDAC) activity and c-myc expression to activate p21 and downregulate cyclin D1 and cdk4, resulting in cell growth arrest and apoptosis on colon cancer [52]; it induced apoptosis and cell cycle arrest by blocking phosphorylation of EGFR pathways, activating JNK pathways and activating p53/p21 pathway to affect cyclin E and cdk2 complex in A549 cancer cells in vitro and in vivo [53]. Azulene have been found the antiproliferation activity on MCF7 breast cancer cells and DU145 prostate cancer cells [54]. As a result, we guessed that patchouli alcohol or azulene might be the major anticancer compound in PPa extract.

**Figure 12:**
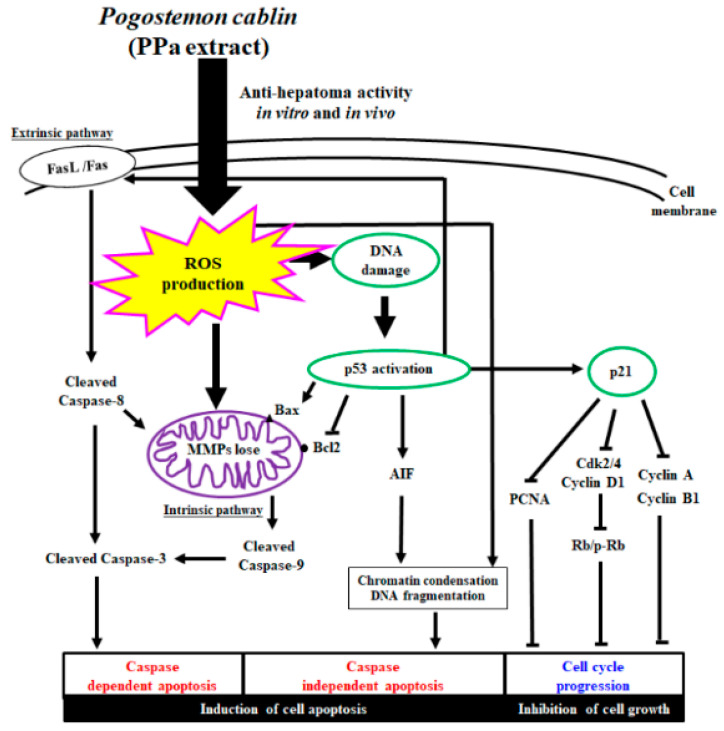
Overview of the mechanisms underlying the anti-hepatoma effects of PPa extract in vitro and in vivo.

In conclusion, PPa extract seems to be a good herbal agent with higher safety margins and effectively suppresses hepatoma by reducing tumor cell growth and inducing tumor cell apoptosis. Moreover, PPa extract plus sorafenib yielded synergistic effects on the AKT/mTOR pathway, reducing cell proliferation and activating the caspase cascade. Thus, in vitro and in vivo data suggest that PPa extract might be an effective anti-hepatoma agent for HCC treatment (Figure 12).

## 4. Materials and Methods

### 4.1. Extration Essential Oils of Pogostemon Cablin (PPa Extract)

*Pogostemon cablin* plant had confirmation of identification by Professor Han-Ching Lin (Department & Graduate Institute of Pharmacology, National Defense Medical Center, Taiwan). The fresh leaves of *Pogostemon cablin*, which was origin from England (2.0 kg) were dried at temperature of 30 °C for 7 h/day for 3 days. After that, *Pogostemon cablin* essential oils was produced by using steam distillation. The dried leaves of *Pogostemon cablin* (500 g) was placed in a 2-L steam distillation steel apparatus unit with a flow rate of generated steam approximately 7.2 mL/min at 100 ℃ for 100 min and the yields were about 2.32% [20]. *Pogostemon cablin* extract (PPa extract) was commissioned to Phoenix (Red Bank, NJ, USA) for large scale extraction. After extraction, the PPa extract was sealed in a black glass bottle and stored at 4 °C. For long-term preservation, avoiding moisture and light was necessary. Before experiments conducting, the concentration of PPa extract was calculated as the equation: the weight of 20 μL PPa extract (g)/ (the weight of 180 μL DMSO + the weight of 20 μL PPa extract) (g) and the final concentration of DMSO in cells was less than 1%.

### 4.2. Cell Culture

HepG_2_, Mahlavu, J5, Huh-7, SVEC, MDCK and BNL CL.2 cell lines were purchased from American Type Culture Collection (Manassas, VA, USA) or Bioresource Collection and Research Center (Hsinchu, Taiwan). The cells were cultured in DMEM or RPMI-1640 supplemented with 10% FBS (Gibco, Mexico), 1% sodium pyruvate, 1% HEPES and 1% penicillin/streptomycin at 37 °C in a humidified incubator supplemented with 5% carbon dioxide. All cell culture reagents were purchased from Gibco/Thermo Fisher Scientific (Waltham, MA, USA). HepG_2_ and Mahlavu cells were analyzed with a Femtopath TP53 Exon8 Primer Set (HongJing Biotech., New Taipei City, Taiwan) to confirm their TP53 levels.

### 4.3. Cell Viability

HCC cells (5 × 10^3^/100 μL), SVEC, MDCK (1 × 10^4^/100 μL), and BNL CL.2 cells (4 × 10^4^/100 μL) were seeded in a 96-well plate. After incubating overnight at 37 ℃, the cells were treated with serially diluted drug concentrations for 24, 48, and 72 h. The cell viability was measured using an MTT assay [20].

### 4.4. Cell Cycle Analysis

Cells (2 × 10^6^) were seeded in 10 cm dishes and were treated with indicated concentration of PPa extract for the indicated time points, and then incubated with PI stain (40 μg/mL) after harvesting the cells. Fluorescence was detected by a FACScan flow cytometer (FASCS Calibur, Franklin Lakes, NJ, USA) and the cell cycle distribution was analyzed by FlowJo 7.6.1 (Ashland, OR, USA) [20].

### 4.5. TUNEL Assay

A TUNEL in situ cell death detection kit (Roche Applied Science, ON, Canada) was used according to the manufacturer’s instructions. Cells (2 × 10^5^) were seeded in 6-well and were treated with PPa extract (20 or 30 μg/mL) for 24 h. After treatment, cells were harvested by 0.05% trypsin, fixed with 10% buffered formalin for 10 min, and smeared on the slides to incubate with 3% H_2_O_2_ for 5 min and 0.1% Triton X-100 for 1 min. Then, TUNEL reaction solution was added to incubate for 2 h and stained with 10 μg/mL propidium iodide (PI, red) for 10 min as counterstain. The average number of TUNEL-positive cells (green) was determined from ten fields under 400× magnification by microscope (ZEISS Axio Imager A2, Bremen, Germany).

### 4.6. Western Blot Analysis

Cells (2 × 10^6^) were seeded in 10 cm dishes and treated with PPa extract (20 or 30 μg/mL) for 0, 6, 12, 24, and 48 h to detect cell proliferative, cell cycle related and apoptotic protein expressions. To verify whether PPa extract activated the caspase cascade, cells (2 × 10^6^) were seeded in 10 cm dishes overnight and pretreated with caspase-3 inhibitor (CPI-370, Z-DEVD-FMK, 1 μM), capase-8 inhibitor (CPI-008, Z-LETD-FMK, 1 μM) or caspase-9 inhibitor (CPI-009, Z-LEHD-FMK 1 μM), which were purchased from G-Biosciences (Louis, MO, USA), for 1 h. After removing caspase inhibitors, PPa extract (20 or 30 μg/mL) were added and incubated for 24 h to detect indicated protein expressions. After getting end points of experiment, the cells were harvested and had cell lysate by RIPA buffer. After cell lysate colleting, protein concentration was quantified using the BCA Protein Assay Reagent (Thermo Fisher Scientific, Waltham, MA USA). Whole cell lysates were fractionated via 8–12% SDS-PAGE and transferred to PVDF membranes by electroblotting. The membranes were blocked with skim milk and were incubated at 4 °C overnight with each primary antibody. The membranes were then incubated with the appropriate secondary antibody and horseradish peroxidase, which were purchased from Santa Cruz Biotechnology (Dallas, TX, USA). Detection was carried out using a T-Pro LumiFast plus Chemiluminescence Detection Kit (T-Pro Biotechnology, New Taipei County, Taiwan). Bands were detected and photographed by a fluorescence imaging analyzer (GE LAS-4000, Little Chalfont, United Kingdom) and were quantified using ImageJ software (NIH, Bethesda, MD, USA). These experiments were independently achieved duplicated. Primary antibodies, including p53 (SC-6243), p-p53 (Ser392; SC-7997), Rb (SC-7905), p-Rb (Ser24/Thr252; SC-16671), p21 (SC-397), PCNA (SC-7907), CDK2 (SC-163), CDK4 (SC-260), cyclin A (SC-751), FAS (SC-715), FASL (SC-834), caspase-8 (SC-5263), caspase-9 (SC-7885), AIF (SC-9416), caspase-3 (SC-98785), AKT (SC-8312), p-AKT (Ser473; SC-7985-R), mTOR (SC-8319), p-mTOR (Ser2448; SC-101738), P70S6K (SC-8418), p-P70S6K (Ser411; SC-8416), ERK (SC-154), p-ERK (Tyr204; SC-7383), and Actin (SC-47778), were purchased from Santa Cruz Biotechnology (Dallas, TX, USA). The primary antibodies of bcl2 (IR94-392), bax (IR93-390), cyclin D1 (IR117-294) and cyclin B1 (IR116-289) were purchased from iReal Biotechnology Co., Ltd. (Hsinchu, Taiwan).

### 4.7. Detection of Reactive Oxygen Species (ROS)

HCC cells were subcultured in a 6-well plate at a density of 1 × 10^5^ cells/mL and were treated with PPa extract (20 μg/mL) at different time points. After collecting cells, ROS generation was assessed using DCFH-DA staining according to the manufacturer’s instructions. FACScan flow cytometry was performed and the data were analyzed by FlowJo 7.6.1.

### 4.8. Mitochondrial Membrane Potential (MMPs, ΔΨm) Assay

Cells were subcultured in a 6-well plate at a density of 1 × 10^5^ cells/mL and then were treated with PPa extract (20 μg/mL) for the indicated times. After the cells harvesting, cells stained with JC-1 probe (AAT Bioquest, Inc., Sunnyvale, CA, USA) according to the manufacturer’s instructions. FACScan flow cytometer was performed and the data were analyzed by FlowJo 7.6.1. The ΔΨm fluorescence was observed and photographed under a microscope at a magnification of ×400 (ZEISS Axio Imager A2, Bremen, Germany).

### 4.9. Synergistic Effect of Ppa Extract Plus Sorafenib on Cell Proliferation and Regrowth

For combined treatment with PPa extract and sorafenib, MTT assay was performed and the data were converted to a readout of fraction of inhibition affected by the individual drug or the combination and analyzed using the combination index method [51,52]. Cells (5 × 10^3^/100 μL) grown in 96-well overnight and treated with as the following for 24 and 48 h: PPa extract (0, 1.9, 3.8, 7.5, 15 and 30 μg/mL) plus sorafenib (2 μg/mL), or sorafenib (0, 0.16, 0.32, 0.64, 1.25 and 2.5 μg/mL) plus PPa extract (15 μg/mL) for HepG_2_ cells; PPa extract (0, 3.2, 6.4, 12.5, 25 and 50 μg/mL) plus sorafenib (3 μg/mL), or sorafenib (0, 0.64, 1.25, 2.5, 5 and 10 μg/mL) plus PPa extract (25 μg/mL) for Mahlavu cells. After end points, the combination index (CI), which was calculated as the equation: CI = IC_50_ of synergistic treatment I/IC_50_ of PPa extract + IC_50_ of synergistic treatment II/IC_50_ of sorafenib. CI values significantly less than 1.0 indicated synergy. For analysis of sub-G_1_ phase, cells (2 × 10^6^) seeded at 10 cm dishes and treated with as following for 24 and 48 h: 2 μg/mL sorafenib plus 15 μg/mL PPa extract for HepG_2_ cells; 3 μg/mL sorafenib plus 25 μg/mL PPa extract for Mahlavu cells. After that, both cell lines were analyzed for flow cytometry. For regrowth assay, cells (5 × 10^2^) seeded at 96-well overnight and treated with PPa extract (15 μg/mL) and sorafenib (0.2 μg/mL) for 4 and 8 days. During treatments, fresh drugs were changed every two days and harvested the data at 4 and 8 days. After treatment, cells were stained with 0.1% crystal violet and absorbance was measured at 550 nm after dissolving in 0.5% acetic acid. For detection of indicated protein expressions, cells (2 × 10^6^) seeded at 10 cm dishes. And then, HepG_2_ cells were treated with 2 μg/mL sorafenib plus 15 μg/mL PPa extract and Mahlavu cells were treated with 3 μg/mL sorafenib plus 25 μg/mL PPa extract for 48 h. Cell lysates were collected for western blot.

### 4.10. Xenograft Animal Study

Balb/c nude mice (8–12 weeks, female) purchased from National Laboratory Animal Center (Taipei, Taiwan) were housed in a pathogen-free environment. All procedures of the liver cancer cell xenograft animal model were performed in the Laboratory Animal Center of Chung Shan Medical University (CSMU) following the Guide for the Care and Use of Laboratory Animals and were approved by the IACUC of CSMU (CSMU-IACUC-1662). To establish a subcutaneous liver cancer model in mice, HepG_2_ cells (1 × 10^6^ cells/100 μL/mouse) were injected into the right flank of mice. After 5 days, mice were randomly divided into two groups (n = 5 for each): vehicle treated with mineral oil or subcutaneously treated with PPa extract (200 mg/kg) on left flank of mice once every two days. All mice were observed until the tumor volume was greater than 1500 mm^3^ (L × H×W mm^3^), which was noted as the last survival day. The following analysis was performed by IHC and H&E staining. The slides were assessed by light microscopy at a magnification of ×400. For serum biochemical estimation, blood was collected from the tail vein at 0, 3, 6, 12, and 24 h for analysis of acute toxicity which is determining the short-term adverse effects of a drug when administered in a single dose, or in multiple doses during a period of 24 h [55], after PPa extract (200 mg/kg) treatment. Serum was separated via centrifugation at 3000 rpm for 10 min for examination of blood cells (white blood cell, red blood cell and platelet) and serum biochemistry (ALT, and AST).

### 4.11. H&E and Immunohistochemistry Staining

The colleting tissues were fixed in 10% buffered formalin, embedded in paraffin wax, cut into 4 μm thick sections. These sections were then stained with Mayer’s hematoxylin and eosin Y solution and were observed under a light microscope after staining at ×200 magnification. Indicated protein detection was performed by immunohistochemical analysis. After antigen retrieval, the sections were treated with 10% BSA solution for blocking and 3% H_2_O_2_ for removing of the activity of endogenous enzymes. The primary antibodies, including 8-oxo-dG (bs-1278R, Bioss Antibodies Inc., Woburn, MA, USA.), caspase-3 (SC-98785), PCNA (SC-7907), VEGF (SC-152), VEGFR1 (SC-9029) and VEGFR2 (SC-6251), which were purchased from Santa Cruz Biotechnology (Dallas, TX, USA) were added and incubated at 4 ℃ overnight. Positive cells were detected with HRP-conjugated secondary antibody and visualized using 3,30-diaminobenzidine (DAB) staining. After immunostaining, sections were counterstained with hematoxylin. The experiment was randomly selected and counted ten ×200 field to achieve IHC score, which was evaluated by three experienced pathologists, independently, using quick score = intensity score × positive area score. The intensity scoring criteria: 0, any tumor cells with membrane staining at intensity of no staining; 1, weak staining; 2, moderate staining; 3, strong staining and 4, strongest staining. The percentage of positive area at the ×200 field are scored as 0 = 0%, 1 = 1–20%, 2 = 21–40%, 3 = 41–60%, 4 = 61–80% and 5 = 81–100%. Scoring of PCNA protein expressions were randomly counted the number of positive cells at ten fields under ×400 magnification and the results were presented as a percentage of the number of positive cells. The data were observed and photographed at ×400 magnification by microscope (ZEISS Axio Imager A2, Bremen, Germany).

### 4.12. Gas Chromatography-Mass Spectrometry Analysis

Gas chromatography-mass spectrometry (GC-MS) analyses were performed using an Agilent 7890CB gas chromatograph (AccuTOF-GCx, Jeol, MA, USA) with an Rxi-5MS capillary column (film thickness: 30 m × 0.25 mm × 0.25 μm) that was commissioned to the National Central Taiwan University Office of Research and Development’s Center for Advanced Instrumentation (Hsinchu, Taiwan). The sample was diluted using hexane (1/500), the carrier gas was helium (1 mL/min), and the injector temperature was 300 °C with an injection flow rate of 1 mL/min. Components were identified by comparing their mass spectra with those obtained from authentic samples or spectra of the Wiley/Nist libraries.

### 4.13. Statistical Analysis

All data are presented as the mean ± SD. Statistical significance between groups was determined by Student’s *t*-test. The evaluation of survival rate utilized Kaplan–Meier software. A *p* value < 0.05 was considered statistically significant.

## Figures and Tables

**Table 1:** The IC_50_ of PPa extract and clinical drugs in HCC and normal cells.

Cell Line	Tumor Type	PPa Extract	SOR	VP-16	5-FU
**Hepatocellular Carcinoma Cells**					
**HepG_2_**	Human HCC cell	20.09 ± 2.21 ^a,b^	3.52 ± 1.97	4.73 ± 3.57	2.09 ± 1.63
**Mahlavu**	Human HCC cell	33.29 ± 2.72 ^a,b^	6.07 ± 1.06	4.54 ± 2.17	14.97 ± 3.71
**J5**	Human HCC cell	29.87 ± 3.62 ^a,b^	2.39 ± 1.91	3.01 ± 2.75	18.79 ± 0.91
**Huh7**	Human HCC cell	7.34 ± 3.09 ^a,b^	1.77 ± 4.31	5.11 ± 2.08	4.88 ± 3.37
**Normal cells**					
**SVEC**	Mouse vascular endothelial cell	69.68 ± 4.63 ^c^	8.55 ± 2.73	1.85 ± 0.49	1.69 ± 2.8
**MDCK**	Canine epithelial kidney cell	73.61 ± 0.16 ^c^	6.95 ± 1.45	3.38 ± 0.43	8.74 ± 0.53
**BNL CL.2**	Mouse liver embryonic cell	147.24 ± 7.71 ^c^	10.75 ± 5.17	6.43 ± 3.02	>20

Note: Values are the mean ± SD (μg/mL) at 48 hr. ^a^: HCC cells were significantly different from normal cells in the PPa extract treatment group (*p* < 0.05). ^b^ and ^c^: PPa extract treatment was significantly different from SOR treatment in both HCC and normal cells (*p* < 0.05). SOR: sorafenib. VP-16: Etoposide. 5-FU: 5- Fluorouracil.

**Table 2:** Comparison of Selectivity index (SI) on drugs.

Normal Cells	/Tumor Cells	PPa Extract	SOR	VP-16
SVEC	/HepG	3.5	2.4	0.4
	/Mahlavu	2.1	1.4	0.4
	/J5	2.3	3.6	0.6
	/Huh7	9.5	4.8	0.4
MDCK	/HepG_2_	3.7	2.0	0.7
	/Mahlavu	2.2	1.1	0.8
	/J5	2.5	2.9	1.1
	/Huh7	10	3.9	0.7
BNL CL.2	/HepG_2_	7.4	3.1	1.4
	/Mahlavu	4.4	1.8	1.4
	/J5	4.9	4.5	2.1
	/Huh7	20.1	6.1	1.3

Note: Selectivity index (SI) = IC_50_ of normal cells/IC_50_ of HCC cells. SI > 2: indicated that drugs have high selectivity for tumor cells; SI < 2: indicated that drugs have poor selectivity for tumor cells [21]. SOR: sorafenib. VP-16: Etoposide.
